# From the G-spot to the H-zone (Haddad Zone): Exploring an Anatomical, Neurovascular, and Regenerative Framework for Female Sexual Function

**DOI:** 10.7759/cureus.114150

**Published:** 2026-08-07

**Authors:** Rashad Haddad

**Affiliations:** 1 Obstetrics and Gynecology/Cosmetic and Regenerative Gynecology, World Academy of Regenerative and Aesthetic Gynecology, Dubai, ARE

**Keywords:** anterior vaginal wall, exosomes, female sexual function, h zone, neurovascular anatomy, orgasmic dysfunction, prp, regenerative gynecology

## Abstract

The anatomical existence of the Gräfenberg Spot (G-spot) remains unverified, and emerging neurovascular research suggests that female sexual responsiveness arises from a broader, continuous anterior vaginal region. This functional area, now defined as the Haddad Zone (H-zone), exhibits dense mechanosensory, vascular, and connective-tissue integration. Regenerative biologics such as platelet-rich plasma (PRP), autologous extracellular vesicles (EVs), mesenchymal stromal cell-derived exosomes, and plant-derived nanovesicles exhibit molecular activity associated with sexual arousal, lubrication, and mucosal health.

This review aimed to explore the anterior vaginal wall as an integrated anatomical and neurovascular region and to propose a conceptual framework for understanding regenerative biologic therapies targeting the H-zone.

This narrative review and conceptual framework article synthesizes published evidence on female sexual anatomy, anterior vaginal wall neurovascular physiology, the historical G-spot concept, and emerging regenerative biologic therapies. Relevant literature on platelet-rich plasma, extracellular vesicles, mesenchymal stromal cell-derived exosomes, hyaluronic acid-based interventions, vaginal tissue biology, and sexual-function outcomes was reviewed to develop a hypothesis-generating model of the H-zone. The procedural discussion is presented as an anatomically informed conceptual rationale and should not be interpreted as prospective procedural validation. No original randomized clinical trial, laboratory analysis, procedural validation study, or human-subject intervention was conducted.

The reviewed literature suggests that the anterior vaginal wall may function as a continuous erogenous region rather than a discrete focal structure. Regenerative biologics appear to influence pathways such as vascular endothelial growth factor (VEGF), Phosphoinositide 3-kinase/Protein Kinase B (PI3K/Akt), extracellular signal-regulated kinase (ERK), transforming growth factor-beta 1 (TGF-β), and hypoxia-inducible factor-1 alpha (HIF-1α), which are relevant to angiogenesis, epithelial restoration, extracellular matrix remodeling, neurosensory responsiveness, and lubrication. In contrast, mechanical augmentation, such as focal hyaluronic acid bulking, primarily provides tissue projection and does not directly address these biological pathways.

The H-zone offers a biologically plausible anatomical and neurovascular framework for understanding female sexual function beyond the traditional focal G-spot concept. Regenerative biologic therapies may influence pathways relevant to vascularity, epithelial integrity, extracellular matrix remodeling, lubrication, and neurosensory responsiveness; however, current evidence remains preliminary and largely indirect. Further controlled clinical and translational studies are required before whole-zone regenerative therapy can be considered an established or superior treatment approach.

## Introduction and background

Female sexual function is a multidimensional process involving neurovascular, endocrine, psychological, and connective-tissue mechanisms that work in concert to generate desire, arousal, orgasm, and resolution. The female sexual response is influenced by the structure and function of the genitalia, hormonal milieu, and central processing of erotic stimuli, consistent with the Dual Control Model of sexual response [1]. Orgasm represents the apex of this physiological and psychological process. It may be elicited through clitoral stimulation, vaginal stimulation, or stimulation of deeper periurethral and anterior vaginal tissues, although the pathways vary substantially among individuals [1].

Historically, attempts to localize female sexual responsiveness to a discrete anatomical site resulted in the popularization of the Gräfenberg Spot (G-spot). Early descriptions suggested a sensitive region on the anterior vaginal wall capable of evoking heightened arousal. However, anatomical and histological investigations have produced conflicting and often contradictory findings. Some studies identified suburethral structures that they proposed corresponded to the G-spot [2], while systematic reviews concluded that the concept lacks consistent anatomical evidence and remains controversial [3]. The reported location, depth, and even existence of the G-spot vary widely across studies, and many women do not experience vaginal orgasmic response through focal stimulation alone, reinforcing the anatomical limitations of the G-spot hypothesis.

Modern neuroanatomical and sensory-mapping studies suggest a broader model. The anterior vaginal wall contains dense innervation and subepithelial vascular plexuses.

Based on this broader understanding, the present review proposes the H-zone as a conceptual anatomical-functional framework encompassing the anterior vaginal wall rather than a discrete anatomical structure. It describes a hypothesized region of distributed neurovascular and mechanosensory responsiveness that may function in concert with the clitoral-urethral-vaginal complex. This framework is intended to integrate available evidence concerning anterior vaginal wall innervation, vascularity, and individual variation in sexual responsiveness [2-6]. It should not be interpreted as an established or independently validated anatomical entity.

Parallel advances in regenerative medicine, including platelet-rich plasma (PRP), extracellular vesicles, mesenchymal stem cell-derived exosomes, and other biomolecules, have introduced new mechanisms for enhancing lubrication, tissue quality, vascularity, and neurosensory responsiveness. PRP delivers growth factors involved in angiogenesis and tissue repair [7], while exosomes participate in gene regulation, collagen remodeling, and cellular communication [8]. Stem cell-derived biologics show promise for restoring epithelial integrity and modulating inflammatory pathways [9]. These therapies act on underlying tissue biology rather than producing mechanical projection, making them conceptually aligned with the H-zone framework rather than the traditional G-spot model.

Accordingly, this manuscript integrates historical, anatomical, neurovascular, and regenerative-biologic evidence to establish a comprehensive scientific framework for female sexual function. It further evaluates the rationale for whole vaginal wall biologic therapy as a mechanism-driven approach that advances beyond the limitations of focal G-spot augmentation. 

In this manuscript, the H-zone is proposed as a conceptual anatomical-functional framework rather than an established discrete anatomical structure. The term is used to describe a hypothesized region of anterior vaginal wall sensitivity based on currently available anatomical, neurovascular, mechanosensory, and regenerative-biologic evidence.

## Review

Anatomy, physiology, and neurovascular biology

Clitoral-Urethral-Vaginal Anatomy

**Figure 1:**
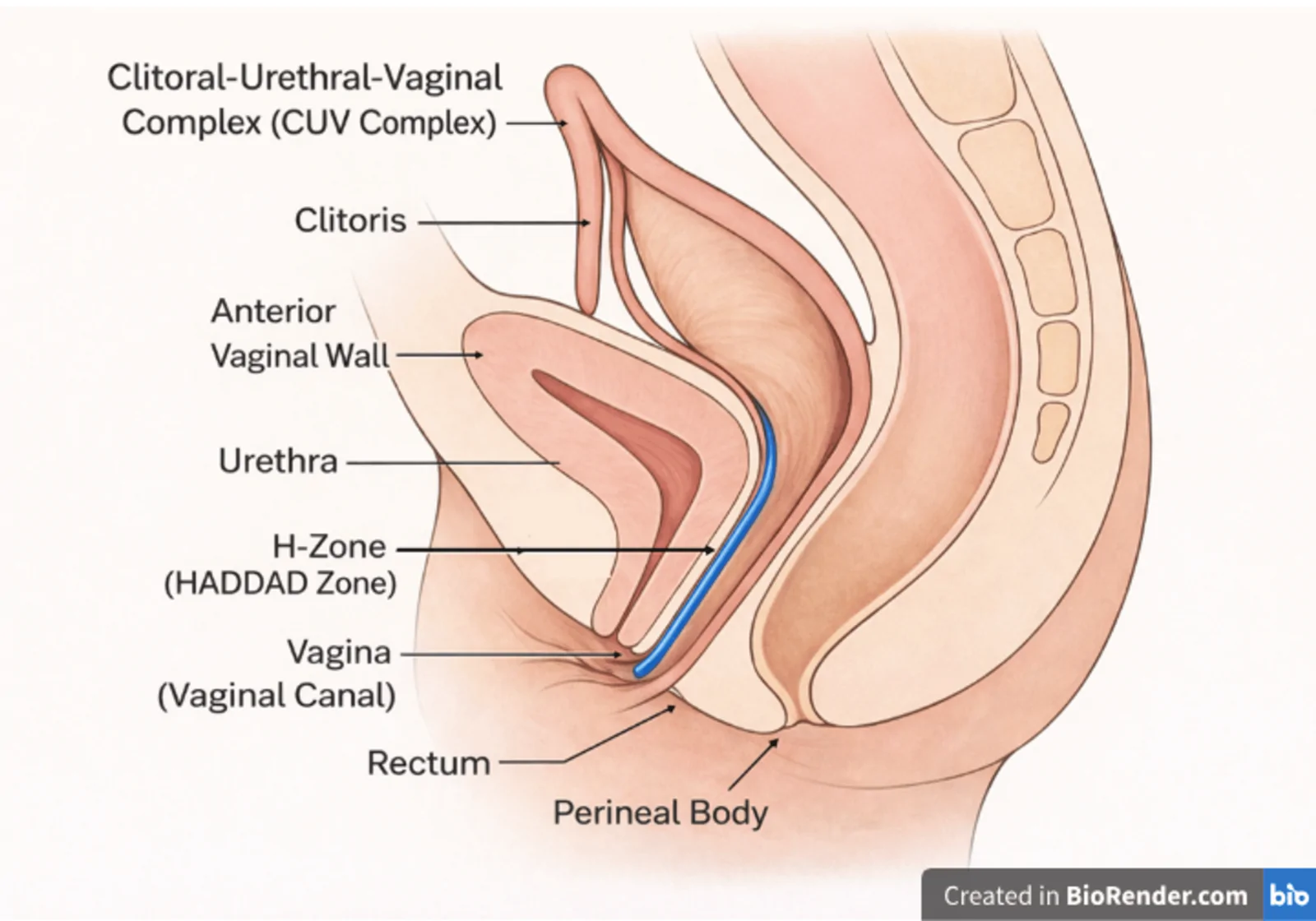
Neurovascular Anatomy of the H-Zone This sagittal schematic illustrates the clitoral-urethral-vaginal (CUV) complex, demonstrating the anatomical relationships between the clitoris, urethra, anterior vaginal wall, vagina, rectum, and perineal body. The H-Zone is depicted as a longitudinal region along the anterior vaginal wall, situated between the urethra anteriorly and the vaginal lumen posteriorly. This zone represents a functionally significant area characterized by close approximation of urogenital tissues and neurovascular structures, which may contribute to vaginal sensitivity and sexual responsiveness. The illustration emphasizes the integrated anatomy of the anterior vaginal wall within the pelvic floor rather than a discrete organ. Image credit: Dr Rashad Haddad. Figure created using BioRender.com (BioRender, Toronto, Canada).

The clitoral complex is the principal sensory organ of sexual arousal and is structurally comparable to the glans penis. It consists of the glans, body, paired crura, and vestibular bulbs, all composed of erectile tissue that becomes engorged during arousal [3]. The glans clitoris, the externally visible component, is densely innervated and highly vascular, allowing rapid vasocongestion in response to sexual stimulation. The integrated clitoral-urethral-vaginal architecture and its neurovascular continuity are illustrated in Figure 1.

The vagina is a fibromuscular canal lined with non-keratinized stratified squamous epithelium and supported by an extensive vascular network [4]. The anterior vaginal wall is thinner and more richly innervated than the posterior wall, containing the urethrovaginal fascia, paraurethral tissues, and subepithelial vascular plexuses that contribute to tactile sensation and lubrication.

The historical concept of the G-spot proposed a discrete erogenous structure located within this anterior vaginal region; however, anatomical studies have failed to identify a consistent or reproducible structure corresponding to a focal organ [2,3]. Instead, emerging evidence demonstrates that the region contains a distributed neurovascular and mechanosensory system, consistent with the concept of a contiguous erogenous zone rather than a single point.

Neurovascular Supply

Blood supply to the vulva and vagina derives primarily from the internal pudendal artery, a branch of the internal iliac artery, with additional supply to the labia majora from the superficial external pudendal artery [4]. Venous drainage occurs through the internal and external pudendal veins.

The pudendal nerve provides innervation to the external genitalia and vaginal wall, arising from sacral roots S2-S4. It divides into branches, including the dorsal nerve of the clitoris, the perineal nerve, and the inferior rectal nerve, supplying sensory and motor fibers to the vulva, clitoris, pelvic floor muscles, and perineum [4]. Autonomic innervation of the clitoral erectile tissue arises from the cavernous nerves of the uterovaginal plexus, contributing to vasodilation and engorgement during arousal.

These neural networks explain why erotic sensitivity is widely distributed across the anterior vaginal region rather than concentrated in a single point. Sensory mapping studies involving 441 sexually active women demonstrated significant heterogeneity in erogenous responses, with the superficial anterior vaginal wall showing the most essential sensory variation [7], supporting the broader H-zone concept.

Physiology of Arousal

Erotic cognition, emotional stimuli, or direct tactile stimulation of the genitalia may initiate sexual arousal. Reflexive arousal pathways integrate sensory input at the sacral spinal cord, producing physiological changes independent of conscious control [5]. Nitric oxide (NO), released by parasympathetic nerve terminals, activates guanylyl cyclase and increases cyclic guanosine monophosphate (cGMP), causing smooth muscle relaxation, vasodilation, and engorgement of erectile tissues in the clitoris and vestibular bulbs [6].

Vaginal lubrication occurs through two mechanisms: transudation of plasma from engorged vaginal capillaries and mucus secretion from the greater vestibular (Bartholin’s) glands during parasympathetic activation [5].

Vasoactive intestinal polypeptide (VIP) contributes to smooth-muscle relaxation, complementing the NO-cGMP pathway [6]. Androgens and estrogens modulate vascular tone, epithelial turnover, and lubrication, underscoring the hormonal dependence of genital tissue health.

Mechanosensory Biology of the Anterior Vaginal Wall

The anterior vaginal wall contains a diverse range of mechanoreceptors, including Ruffini endings, free nerve endings, and pressure-sensitive corpuscles, that respond to stretching, pressure, and contact during sexual activity. These receptors project to sacral segments S2-S4, contributing to pleasurable sensations even in the absence of direct external clitoral stimulation.

The region’s sensitivity is further enhanced by rich subepithelial vascularity, high-density autonomic and somatic nerve fibers, and close anatomical integration with the clitoral-urethral-vaginal complex.

This distributed architecture provides a biological explanation for the H-zone, which conceptualizes the entire anterior vaginal wall as a unified functional structure rather than an isolated G-spot. The heterogeneity of sensory responses observed in clinical mapping studies further supports this model [7].

Molecular Regulators of Vaginal Tissue Function

Several molecular pathways play pivotal roles in maintaining the structural and functional integrity of genital tissues. Vascular endothelial growth factor (VEGF) is essential for angiogenesis and microvascular repair, thereby improving tissue perfusion and oxygenation. Transforming growth factor-beta 1 (TGF-β1) regulates extracellular matrix remodeling and collagen turnover, which are critical for maintaining tissue strength and structural support. The phosphoinositide 3-kinase/protein kinase B (PI3K/Akt) and extracellular signal-regulated kinase (ERK) signaling pathways promote epithelial cell proliferation, differentiation, and survival, contributing to mucosal integrity and regenerative capacity. Hypoxia-inducible factor-1 alpha (HIF-1α) mediates adaptive responses to reduced oxygen availability by stimulating vascular growth and enhancing tissue recovery. Additionally, the ratio of collagen types I and III plays a key role in determining tissue elasticity and biomechanical resilience, which are essential for maintaining normal vaginal function and responsiveness.

Declines in estrogen, childbirth-related trauma, inflammatory changes, and aging can disrupt these pathways, contributing to dryness, decreased lubrication, and reduced sexual responsiveness.

Regenerative biologics, including PRP, exosomes, and stem-cell-derived factors, have been shown to activate several of these pathways in gynecologic and reproductive tissues [7-10], forming the scientific basis for whole-vaginal-wall regenerative therapy aligned with the H-zone framework.

Regenerative biology: PRP, exosomes, extracellular vesicles, and stem cells

Platelet-Rich Plasma (PRP)

Platelet-rich plasma (PRP) is an autologous preparation of platelets obtained by centrifugation of peripheral blood, yielding a preparation containing 5-10 times the growth factor levels of baseline serum [8]. PRP contains numerous bioactive mediators, including transforming growth factor-β (TGF-β), fibroblast growth factors (FGFs), insulin-like growth factors (IGF-1 and IGF-2), vascular endothelial growth factor (VEGF), and epidermal growth factor (EGF). These molecules collectively contribute to angiogenesis, cell proliferation, epithelial regeneration, and extracellular matrix (ECM) remodeling [8].

Mechanism of Action: PRP activates multiple molecular pathways implicated in genital tissue health: (1) VEGF signaling enhances microvascular density and mucosal perfusion, (2) TGF-β and FGFs stimulate fibroblast activity and collagen remodeling, (3) PI3K/Akt and ERK pathways support cellular survival, proliferation, and epithelial renewal, (4) anti-inflammatory cytokines promote tissue healing and reduce mucosal irritation.

These mechanisms align directly with the needs of the H-zone, where sexual responsiveness depends on optimal vascularity, lubrication, epithelial hydration, and nerve sensitivity.

Applications in Female Sexual Medicine: PRP has shown promise in the management of female sexual dysfunction, including arousal insufficiency, vaginal dryness, and impaired sensitivity. Studies demonstrate improvements in lubrication, epithelial thickness, vascular perfusion, and subjective sexual satisfaction following PRP administration to the anterior vaginal wall or lower introitus [11].

Thus, PRP provides a biologically coherent intervention for enhancing the functional environment of the H-zone.

Exosomes and Extracellular Vesicles (EVs)

Exosomes are nano-sized extracellular vesicles (30-150 nm) secreted by various cell types and involved in intercellular communication by transporting proteins, mRNA, microRNA, lipids, and bioactive molecules between cells [7]. They play fundamental roles in gene regulation, tissue repair, and modulation of the immune microenvironment.

**Figure 2:**
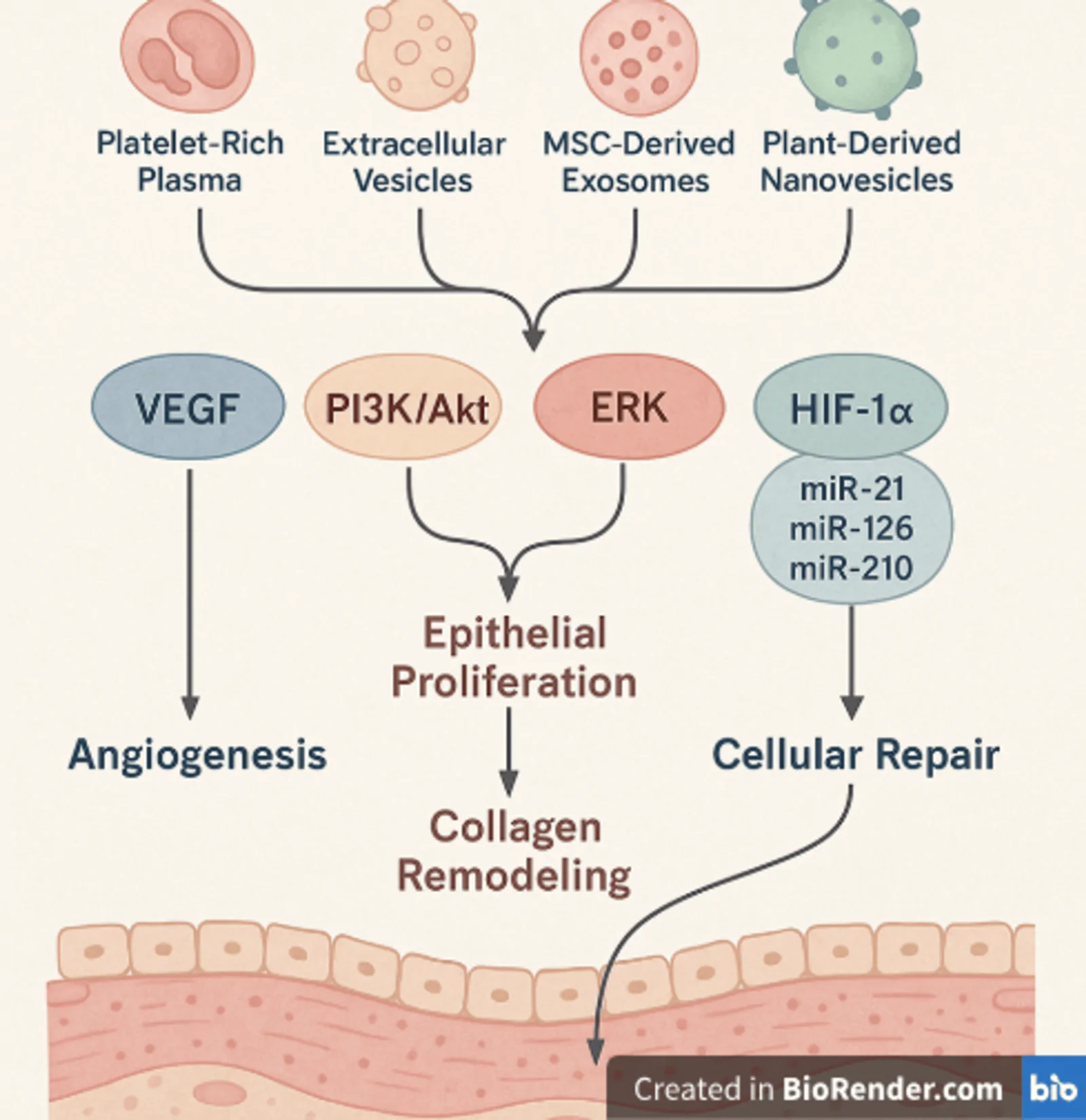
Molecular Pathways Activated by Regenerative Biologics This figure depicts the major intracellular pathways stimulated by platelet-rich plasma, extracellular vesicles, mesenchymal stromal cell–derived exosomes, and plant-derived nanovesicles. The visual representation includes the activation of VEGF pathways that promote angiogenesis, PI3K/Akt and MAPK/ERK pathways that enhance epithelial proliferation and cellular repair, TGF-β signaling that regulates collagen remodeling, and HIF-1α–mediated tissue responses to microvascular hypoxia. MicroRNA activity, particularly involving miR-21, miR-126, and miR-210, is illustrated to demonstrate their regulatory effects on endothelial recovery, inflammation, and extracellular matrix turnover. Together, these mechanisms explain how regenerative biologics may support pathways involved in tissue repair and function. Image credit: Dr Rashad Haddad. The figure was created using BioRender software (BioRender Inc., Toronto, Canada). VEGF: vascular endothelial growth factor, TGF-β: transforming growth factor-beta 1, HIF-1α: hypoxia-inducible factor-1 alpha, miR: microRNA; MSC: mesenchymal stem cell; PI3K/Akt: phosphoinositide 3-kinase/protein kinase B; MAPK/ERK: mitogen-activated protein kinase/extracellular signal-regulated kinase

Molecular Mechanisms: Exosomes contain microRNAs such as miR-21, miR-126, and miR-210, which are implicated in endothelial proliferation, angiogenesis, ECM regulation, modulation of inflammation, and neurotrophic support [7]. These processes directly support vaginal tissue function, lubrication, and neurosensory responsiveness. The primary molecular pathways stimulated by regenerative biologics in vaginal tissues are summarized in Figure 2.

Relevance to Female Reproductive and Vaginal Health: Reproductive tissues, including granulosa cells, follicles, endometrium, and oocytes, naturally release exosomes, which regulate follicular maturation, implantation, embryo development, uterine receptivity, and maintenance of pregnancy [7].

In the context of sexual medicine, adipose-derived mesenchymal stem cell (AD-MSC) exosomes have shown the ability to regenerate vaginal epithelium, strengthen connective tissue, enhance lubrication, increase tightness and sensation, and promote collagen synthesis [12]. Such mechanisms complement the neurovascular physiology of the H-zone and represent an advanced biological approach beyond focal augmentation procedures.

Stem Cells

Stem cells possess the ability to self-renew and differentiate into specialized cell types, making them promising candidates for regenerative therapies [9]. They may be embryonic, fetal, adult, or induced pluripotent stem cells (iPSCs), and their therapeutic capacities derive from both cellular differentiation and paracrine effects.

Mechanisms Relevant to Vaginal and Sexual Function: Mesenchymal stem cells (MSCs), derived from adipose tissue, bone marrow, or other sources, exert regenerative effects through paracrine secretion of growth factors and exosomes, modulation of inflammation, promotion of angiogenesis, and restoration of epithelial and stromal integrity [9].

Clinical Potential: MSC-based therapies have been explored for reproductive disorders such as diminished ovarian reserve and polycystic ovary syndrome (PCOS), where they restore tissue function via molecular and paracrine pathways [9]. Their applicability to vaginal rejuvenation and sexual dysfunction is biologically plausible, given the shared molecular pathways involved in tissue health, vascularity, and ECM dynamics.

When administered to the anterior vaginal wall, these biologic agents may enhance lubrication, increase elasticity, and improve neurosensory responsiveness-all features essential to the functional dynamics of the H-zone.

Integration With the H-zone Model

The regenerative biologics described above - PRP, exosomes, EVs, and MSC-derived products - activate the same signaling pathways (VEGF, PI3K/Akt, ERK, TGF-β, HIF-1α) that maintain the structural and functional health of the anterior vaginal wall. Unlike hyaluronic acid fillers, which offer only mechanical projection, regenerative biologics may support pathways involved in tissue repair and function, aligning perfectly with the neurovascular and mechanosensory architecture of the H-zone [13-16].

This mechanistic synergy underscores why whole-vaginal-wall biologic therapies are conceptually and clinically sounder than traditional G-spot augmentation [14-18].

G-Spot to H-zone framework

From the Historical G-Spot Concept to a Modern Neuroanatomical Framework

The G-spot was historically proposed as a discrete erogenous structure located in the anterior vaginal wall, based on early reports suggesting heightened sensitivity in this region [1]. Anatomical studies, such as those by Ostrzenski, described suburethral structures that were hypothesized to represent the G-spot [2]. The anatomical observations reported by Ostrzenski [2] remain important in the historical evolution of the G-spot concept, particularly because they suggested the presence of a localized suburethral structure within the anterior vaginal wall. However, even if such a structure is present in some individuals, its clinical relevance as the sole target of intervention remains uncertain. Expert clinical observations have suggested that focal injection of this small anterior vaginal wall area with high-molecular-weight hyaluronic acid may produce only temporary mechanical projection and may not consistently improve intimate sensitivity. This supports the need to distinguish between a possible localized anatomical finding and the broader functional network responsible for anterior vaginal wall responsiveness. Accordingly, the H-zone model does not reject prior anatomical observations but expands the interpretation toward a larger neurovascular and mechanosensory region requiring further validation [2]. However, systematic evaluations of the available evidence, including a central review by Vieira-Baptista et al., conclude that the G-spot lacks consistent anatomical identification and remains a subject of scientific controversy [3]. Variability in reported location, depth, and structural morphology across studies further challenges the model of a singular anatomical organ responsible for vaginally mediated orgasm.

Moreover, many women do not achieve orgasm through vaginal penetration alone, and those who do report variable areas of stimulation, suggesting that erotic sensation is not tied to a fixed structure but distributed across a broader region [1]. These limitations highlight the need for a more physiologically accurate framework.

The transition from a focal G-spot model to a zone-based framework is supported by the observation that female genital sensitivity is not uniformly localized to a single reproducible anatomical structure. Instead, available anatomical and sensory data suggest that erotic responsiveness varies across individuals and is influenced by the interaction of the anterior vaginal wall, periurethral tissues, clitoral-urethral-vaginal complex, subepithelial vascular plexuses, and somatic and autonomic nerve supply. Therefore, the proposed H-zone should not be interpreted as a newly identified discrete organ, but as a conceptual anatomical region that integrates multiple adjacent structures contributing to anterior vaginal wall sensitivity. This distinction provides a more cautious and biologically plausible bridge between the limitations of the traditional G-spot hypothesis and the broader functional model proposed in this manuscript.

Sensory Mapping Evidence Supporting a Zone-Based Model

Contemporary sensory-mapping data provide an important rationale for a zone-based interpretation of anterior vaginal wall sensitivity. In a topographic study of 441 sexually active women, Stelmar et al. demonstrated substantial heterogeneity in erogenous sensation across the vulva and vagina, with the superficial anterior vaginal wall showing notable sensory responsiveness and variability [19]. Such variability is difficult to reconcile with a single fixed anatomical point and is more consistent with a distributed field of neurovascular and mechanosensory interaction. These findings do not independently prove the H-zone as a distinct anatomical entity; rather, they support the plausibility of conceptualizing the anterior vaginal wall as a broader functional region involved in sexual responsiveness.

The anterior vaginal wall contains subepithelial vascular plexuses, dense autonomic and somatic nerve fibers, paraurethral connective tissues, and pressure-sensitive mechanoreceptors. All of which participate in sexual arousal, lubrication, and orgasmic response [4,5,6]. This architecture is inherently regional and integrative rather than focal, reinforcing the inadequacy of the G-spot hypothesis.

The H-zone: A Comprehensive Functional Anatomy

The H-zone is proposed in this manuscript as a conceptual anatomical-functional framework for understanding anterior vaginal wall sensitivity. Rather than defining a newly established anatomical organ or discrete structure, the H-zone describes a hypothesized broader region involving the anterior vaginal wall, periurethral tissues, clitoral-urethral complex, vascular plexuses, mechanoreceptors, and somatic and autonomic neural pathways. This framework is intended to integrate existing anatomical and sensory-mapping evidence and to guide future research, rather than to claim definitive anatomical validation.

This zone-based interpretation is also consistent with expert anatomical and procedural observations in which a discrete G-spot structure may not be identifiable during cadaveric dissection, whereas clinical improvement in intimate sensitivity has been observed after platelet-rich plasma administration across multiple sites of the anterior vaginal wall. Although such observations should be interpreted as experiential and hypothesis-generating rather than definitive evidence, they support the rationale for shifting from a focal G-spot model to a broader framework of anterior vaginal wall sensitivity, which requires further anatomical and clinical validation.

Its functional characteristics include high mechanoreceptor density, rich vascularity and rapid vasocongestion, close anatomical integration with the clitoral-urethral-vaginal complex, concentrated sensory afferents from sacral roots S2-S4, and high responsiveness to pressure, shear, and mechanical stretch.

These features collectively produce erotic sensation during penetration and account for interindividual differences in sensitivity and orgasmic patterns.

Rationale for Whole-Wall Stimulation: Biological, Not Mechanical

**Figure 3:**
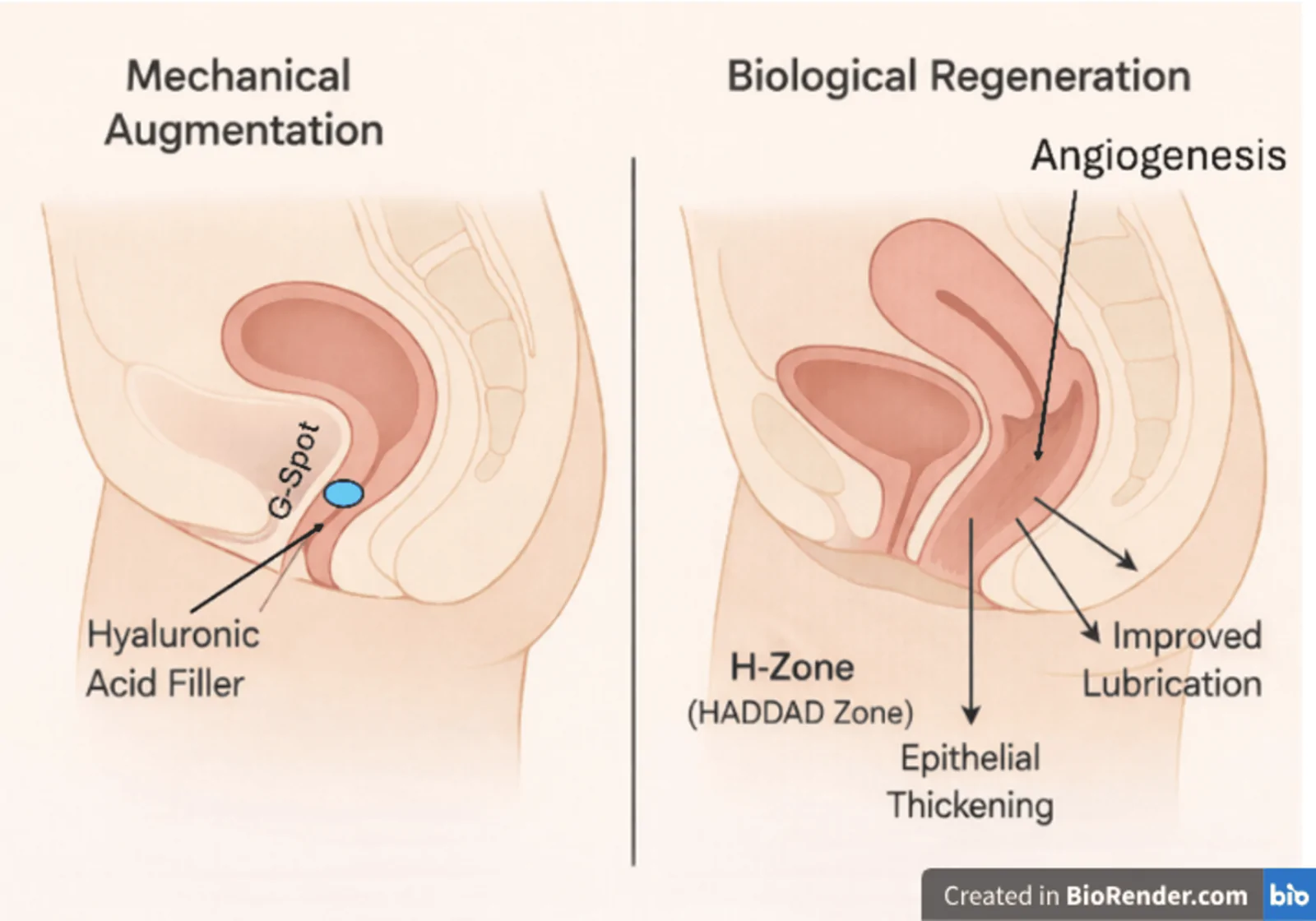
Mechanical Augmentation versus Biological Regeneration These comparative schematic contrasts mechanical augmentation and biological regeneration approaches targeting the anterior vaginal wall. Left panel (Mechanical Augmentation): Localized injection of hyaluronic acid filler produces temporary tissue distension at the level of the anterior vaginal wall, commonly referred to as the G-spot region. This approach primarily results in short-term volumetric enhancement without inducing structural or biological tissue remodeling. Right panel (Biological Regeneration): Regenerative therapy directed toward the H-Zone (Haddad Zone) is shown to promote angiogenesis, epithelial thickening, and improved mucosal lubrication, reflecting restoration of tissue architecture and function. This strategy emphasizes biological remodeling rather than mechanical expansion. Image credit: Dr Rashad Haddad. The figure was created using BioRender software (BioRender, Toronto, Canada).

The historical approach of G-spot augmentation using fillers attempted to create a mechanical projection of the anterior vaginal wall. Therefore, focal hyaluronic acid injection should be interpreted primarily as a localized mechanical augmentation strategy, rather than as a treatment that directly modifies the distributed neurovascular, epithelial, and connective-tissue pathways involved in anterior vaginal wall sensitivity. However, such methods do not target the underlying biological systems responsible for arousal and lubrication. Evidence now shows that sexual responsiveness is mediated by vascular perfusion, epithelial hydration, collagen composition, and neurotransmission, not merely by increased tissue bulk [5,6]. A comparison of localized mechanical augmentation and whole-zone biological regeneration is illustrated in Figure 3.

Therefore, the rationale for whole-wall regenerative therapy is grounded in biological principles rather than mechanical augmentation alone. Tissue vitality, rather than simple tissue volume, plays a central role in maintaining normal sexual function. Regenerative processes such as angiogenesis enhance microvascular perfusion, which improves vaginal lubrication and sensory responsiveness. Extracellular matrix remodeling contributes to improved tissue elasticity, structural integrity, and comfort during sexual activity. In addition, neurotrophic signaling supports nerve function and enhances sensory perception. Regenerative biologics, including platelet-rich plasma, exosomes, and mesenchymal stem-cell-derived products, stimulate these restorative pathways at the cellular and molecular levels. This biological mechanism explains their potential therapeutic advantage over localized filler injections, which primarily provide temporary structural augmentation without addressing the underlying tissue health.

Alignment of Regenerative Therapy With H-zone Physiology

The H-zone model provides a coherent anatomical and physiological guide for targeted interventions. The biological effects of regenerative therapies, including VEGF-driven angiogenesis, TGF-β-mediated collagen remodeling, PI3K/Akt and ERK activation, and enhancement of epithelial integrity, directly match the functional needs of the anterior vaginal wall [7-9].

Unlike focal G-spot augmentation, whole-wall biologic therapy targets the integrated structure and function of the anterior vaginal wall. These therapies support continuous vascular networks, thereby enhancing tissue perfusion and promoting improved lubrication through increased plasma transudation. They also contribute to the restoration of epithelial thickness and integrity, which is essential for maintaining mucosal health and resilience. In addition, regenerative biologics improve collagen composition and balance, resulting in enhanced tissue elasticity and mechanical strength. Furthermore, these therapies promote neurosensory responsiveness by supporting nerve function and tissue regeneration. Collectively, these effects represent a paradigm shift from purely mechanical augmentation toward biologic restoration, aligning closely with the neurovascular and functional foundations of the H-zone.

Conceptual framework

This manuscript employed a hybrid methodological framework integrating structured literature synthesis with translational biological analysis and procedural evaluation. The objective of this approach was to examine the anatomical, neurovascular, and molecular foundations of the H-zone and to contextualize regenerative biologic therapies within a scientifically coherent therapeutic model. A narrative review of the literature was conducted using peer-reviewed studies on anatomical, physiological, neurovascular, and regenerative medicine relevant to female sexual function. Key sources included authoritative descriptions of clitoral and vaginal anatomy [3,4], foundational work on the physiology of sexual arousal [5,6], molecular and cellular studies on extracellular vesicles and exosomes [7], research on platelet-rich plasma (PRP) and its regenerative mechanisms [8,11], and investigations into the therapeutic potential of stem cells and biologic agents in reproductive tissues [9,12, 13]. Sensory-mapping studies were also examined to assess the distribution of erotic sensation across the anterior vaginal wall, particularly the extensive cohort analysis demonstrating significant heterogeneity in sensory responses [14].

To establish the clinical relevance of regenerative therapies in the context of female sexual function, conceptual clinical parameters were derived from existing studies evaluating PRP or biologic injections for sexual dysfunction. Published clinical observations describing improvements in lubrication, arousal, vaginal comfort, and subjective sexual satisfaction after PRP administration to the anterior vaginal wall provided a foundation for defining functional outcome domains [11]. These domains were synthesized to outline a theoretical structure for assessing biologic therapeutic interventions for the H-zone.

A translational biological analysis was conducted to evaluate molecular pathways relevant to tissue regeneration within the anterior vaginal wall. Particular emphasis was placed on angiogenic, proliferative, and extracellular matrix-modulating mechanisms, including VEGF-mediated microvascular enhancement, PI3K/Akt and ERK signaling pathways, and TGF-β-driven collagen remodeling [8]. Studies on exosome-mediated microRNA regulation examined the roles of miR-21, miR-126, and miR-210 in endothelial proliferation, immunomodulation, and extracellular matrix turnover [7,12]. These molecular insights were aligned with established physiological mechanisms of female genital arousal, such as vasocongestion, lubrication via epithelial transudation, and neural responsiveness [5,6], thereby enabling an integrated understanding of how regenerative biologics may enhance H-zone function.

An anatomically informed procedural rationale was conceptualized by examining established descriptions of anterior vaginal wall injection techniques [10,14] and adapting them into a regenerative model consistent with the anatomical boundaries of the H-zone. The method was evaluated with respect to anatomical safety, potential injection depth, mucosal and submucosal deposition patterns, and biological diffusion principles informed by PRP and exosome studies [7,8,12]. The goal was not to replicate a single procedural protocol but to ensure that the therapeutic logic was anatomically coherent and aligned with the contemporary understanding of the anterior vaginal wall as a functional zone rather than a focal structure.

At present, the available literature does not provide sufficient evidence to define an optimal injection depth, biologic dose, injection volume, spacing between injection points, tissue diffusion pattern, or standardized safety profile for H-zone-oriented regenerative therapy. Therefore, the procedural model presented in this manuscript should be interpreted only as a conceptual anatomical framework. It is not intended to function as a clinical protocol or technical recommendation. Future studies should prospectively evaluate injection depth, biologic concentration, dose-response relationships, tissue distribution, diffusion characteristics, vascular safety, adverse events, and durability of clinical response before standardized procedural guidance can be proposed.

**Figure 4:**
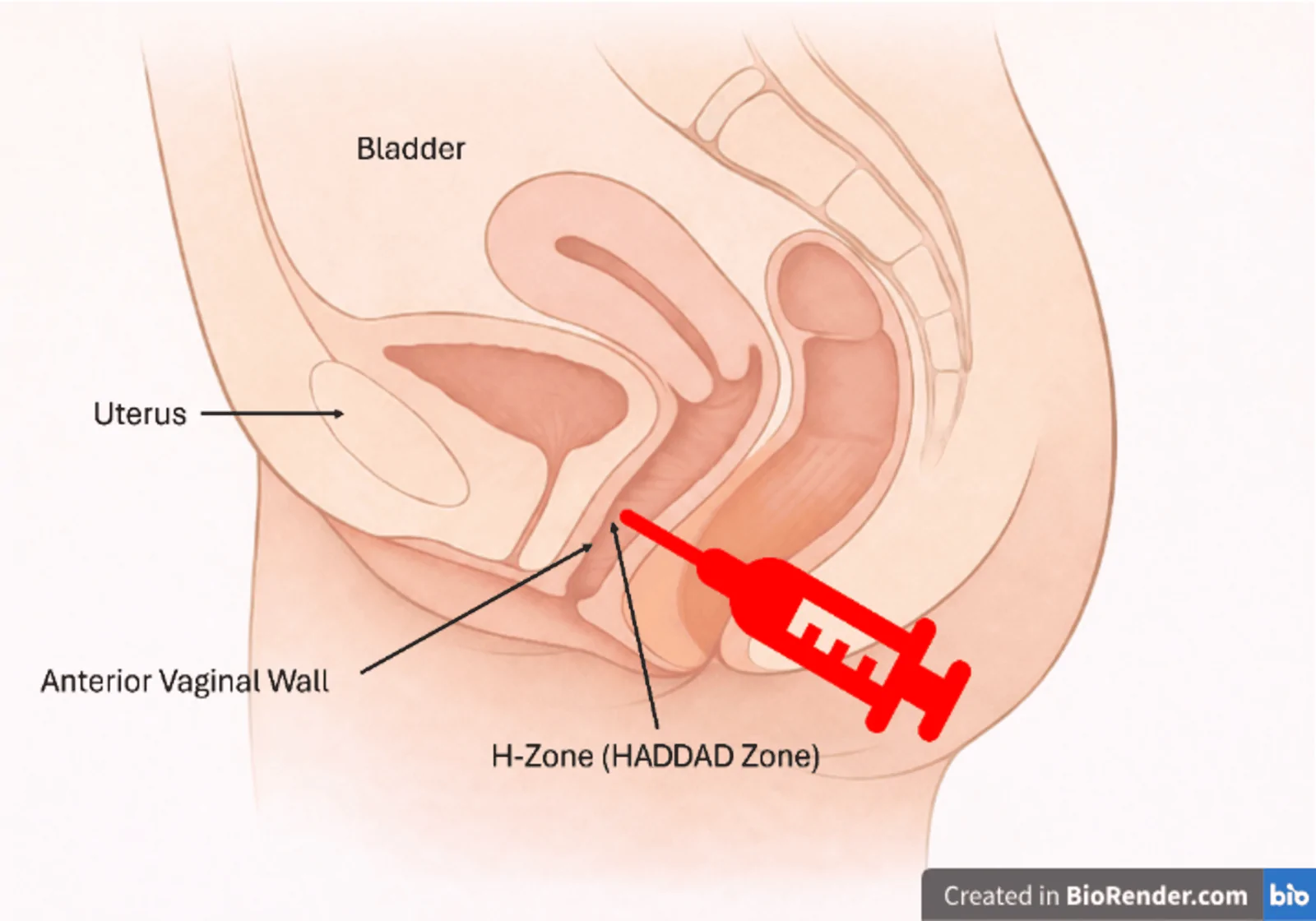
Conceptual Illustration of the H-Zone-Oriented Anterior Vaginal Wall Injection Approach This schematic depicts a conceptual injection strategy targeting the H-Zone (Haddad Zone) along the anterior vaginal wall, shown in relation to adjacent pelvic organs, including the bladder and uterus. This figure does not define an optimal injection depth, dosage, injection volume, diffusion pattern, or validated safety protocol; these parameters require prospective anatomical, imaging-based, and clinical validation. The illustrated approach emphasizes distribution along the length of the anterior vaginal wall rather than focal-point injection, with the intent of engaging the functionally integrated clitoral-urethral-vaginal complex. The figure represents a theoretical framework for uniform tissue engagement and should be interpreted as a conceptual anatomical guide rather than a procedural directive. Image credit: Dr Rashad Haddad. The figure was created using BioRender software (BioRender, Toronto, Canada).

Outcome evaluation domains were derived from published clinical and translational research, emphasizing vascular responsiveness, mucosal integrity, epithelial thickness, lubrication, sensory enhancement, and patient-reported improvement in sexual function. Safety considerations were informed by studies showing favorable tolerability of hyaluronic acid derivatives, PRP, and biologic agents, with no significant evidence of fibrosis or adverse structural changes following localized therapy [15,16]. Because this work is a synthesis of published evidence and does not involve original clinical experimentation, the proposed H-zone model and its therapeutic implications should be interpreted as a hypothesis-generating conceptual framework rather than as a clinically validated treatment paradigm [8,11,17]. A schematic overview of the whole-vaginal-wall biologic injection technique and its hypothesized distribution pattern within the proposed H-zone is shown in Figure 4.

Clinical applications

Historical Approaches to Anterior Vaginal Wall Stimulation

Clinical attempts to enhance vaginal sensitivity have traditionally centered on the concept of localized G-spot augmentation. Hyaluronic acid injections were introduced as a technique to mechanically elevate the anterior vaginal wall, with the intention of increasing friction-based stimulation during intercourse [10]. While some patients reported transient improvement in sexual pleasure, the method fundamentally relied on structural projection rather than biological enhancement. Because hyaluronic acid fillers do not interact with key physiological pathways involved in arousal - such as epithelial hydration, microvascular perfusion, or neurosensory signaling - their effects are inherently limited. Pharmacologic research demonstrates that agents such as papaverine and sildenafil enhance genital blood flow and vaginal lubrication by relaxing smooth muscle and activating the NO-cGMP pathway [6]. Hyaluronic acid, however, does not participate in these vascular mechanisms and, therefore, cannot improve the underlying tissue health required for sustained sexual responsiveness.

Transition Toward Regenerative Biologic Therapies

Growing recognition of the complexity of female sexual physiology has driven a shift from mechanical augmentation toward regenerative biological approaches. Platelet-rich plasma (PRP) has become one of the most widely explored modalities because it delivers concentrated autologous growth factors that can stimulate angiogenesis, epithelial repair, and extracellular matrix remodeling [8]. Clinical observations suggest that PRP injection into the anterior vaginal wall improves lubrication, mucosal resilience, and subjective sexual satisfaction, reflecting its capacity to enhance the biological foundations of arousal rather than merely altering tissue volume [11]. The regenerative action of PRP aligns with the anatomical and neurovascular characteristics of the H-zone, where sexual function depends on tissue vitality, vascular perfusion, and neurosensory responsiveness.

Exosomes and Extracellular Vesicles in Vaginal Tissue Regeneration

Exosomes and extracellular vesicles have emerged as advanced biologic agents with substantial therapeutic potential in sexual medicine. These nanoscale vesicles deliver microRNAs, proteins, lipids, and signaling molecules that regulate inflammation, angiogenesis, collagen turnover, and epithelial proliferation [7]. Exosomes derived from mesenchymal stem cells have been shown to support connective-tissue integrity, enhance lubrication, improve elasticity, and promote epithelial healing through paracrine signaling pathways [12]. Because these biologic properties directly influence determinants of sexual arousal, particularly lubrication, sensitivity, and mucosal comfort, exosomes offer a mechanistically distinct and potentially advantageous approach compared with focal augmentation techniques that lack regenerative effects.

Whole-Wall Regeneration and the H-zone Concept

The transition from focal G-spot injections to whole-anterior-wall biologic stimulation reflects a deeper understanding of the erogenous structure of the vagina. Evidence indicates that sexual responsiveness arises from a distributed neurovascular and mechanosensory network rather than a discrete point [18-19]. The H-zone framework positions the entire anterior vaginal wall as an integrated functional unit containing dense vascular and neural structures essential for arousal. Regenerative biologic therapies, whether PRP, exosomes, or stem-cell-derived secretomes, activate molecular pathways that enhance epithelial hydration, collagen remodeling, vascular perfusion, and neurosensory activity. These processes mirror the physiological mechanisms responsible for sexual function, making whole-wall regenerative therapy a more anatomically and biologically coherent intervention than localized filler-based augmentation.

Safety, Patient Selection, and Clinical Considerations

**Figure 5:**
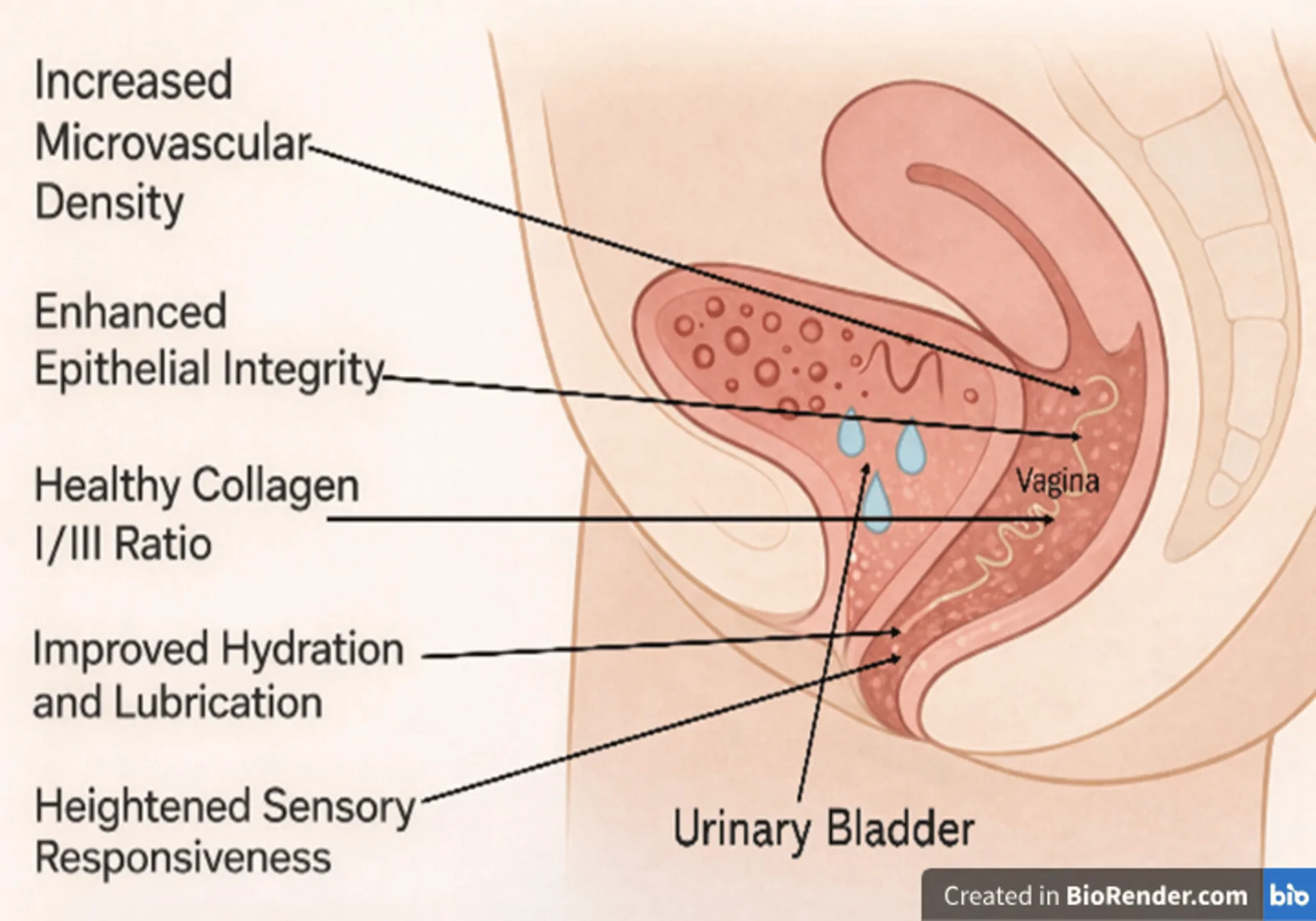
Hypothesized Regenerative Outcomes in The H-Zone This schematic illustrates the anticipated structural and functional tissue changes associated with biologic regeneration targeting the H-Zone along the anterior vaginal wall. The depicted outcomes include increased microvascular density, enhanced epithelial integrity, restoration of a physiological collagen type I/III ratio, and improved tissue hydration and lubrication. Collectively, these changes are hypothesized to contribute to heightened sensory responsiveness of the vaginal mucosa. The figure represents conceptual regenerative effects based on tissue biology principles rather than direct clinical outcome measures. Image credit: Dr Rashad Haddad. The figure was created using BioRender software (BioRender, Toronto, Canada).

Available studies indicate that regenerative biologic therapies, when administered appropriately, demonstrate favorable safety profiles with minimal risk of fibrosis, infection, or adverse mucosal reactions [15,16]. Ideal candidates are women experiencing decreased lubrication, diminished vaginal sensitivity, discomfort during intercourse, or orgasmic difficulties associated with tissue atrophy or neurovascular insufficiency. Because biologic agents act locally and promote tissue-level restoration, they avoid systemic side effects and are generally well tolerated. The alignment between regenerative mechanisms and H-zone physiology supports their potential to improve sexual satisfaction, relationship well-being, and overall quality of life. The anticipated histological and functional improvements following regenerative biologic therapy are depicted in Figure 5.

Future directions

Current Gaps in Knowledge

Despite growing interest in regenerative approaches for enhancing female sexual function, significant gaps remain in understanding their long-term efficacy, safety, and mechanism of action. Existing clinical studies are few in number, frequently involve small sample sizes, and vary in methodology, making it challenging to establish standardized protocols or broadly applicable outcome measures [16]. The field of sexual medicine suffers from a wider historical limitation: research has disproportionately focused on male sexual function, leaving many aspects of female physiology underexplored. Studies of topical agents, vasodilators, and mucosal therapies have shown potential benefits, yet a lack of Class A clinical trials underscores the need for more rigorous investigation [16]. Moreover, the relatively recent emergence of biomolecule-based therapies means long-term follow-up data are limited, and extended safety evaluations are essential before these interventions can be fully integrated into routine practice.

The therapeutic challenge is compounded by the multifactorial nature of female sexual dysfunction, which involves hormonal, psychological, relational, vascular, neurologic, and structural factors [1]. While regenerative biologics offer a promising tissue-focused solution, they address only part of a broader spectrum of influences on sexual well-being. Complementary research is needed to clarify how these therapies interact with hormonal status, pelvic floor function, psychological distress, and other determinants of sexual satisfaction.

Research Priorities and Technological Innovation

Future studies must expand beyond preliminary observational data and adopt more rigorous frameworks that include randomized controlled trials, standardized injection protocols, validated outcome measures, and defined follow-up intervals. Optimizing biologic formulations - such as concentration, preparation techniques, and delivery modalities - will be critical to achieving reproducible therapeutic outcomes. Attention should also be directed toward improving our understanding of vaginal tissue biomechanics, mucosal immunology, and neurovascular regulation, all of which directly impact responsiveness within the H-zone [20-22].

Emerging pharmaceutical technologies offer an opportunity to enhance vaginal drug delivery. Many existing formulations suffer from rapid washout, insufficient mucosal residence time, variable dispersion, and inconsistent therapeutic absorption due to changes in vaginal pH, fluid composition, and hormonal cycling [23]. Improving mucoadhesive properties, designing intelligent polymers responsive to physiological conditions, and developing controlled-release vehicles will significantly improve local therapeutic precision. Such innovations will be invaluable for administering regenerative agents, ensuring sustained bioavailability within the anterior vaginal wall.

Long-Term Outcomes and Mechanistic Evaluation

Long-term studies are essential to determine the durability of regenerative effects on lubrication, mucosal elasticity, sexual sensitivity, and orgasmic function. Preclinical and clinical data already suggest that hyaluronic acid derivatives and other biologically active molecules can improve vaginal hydration, reduce dyspareunia, and enhance epithelial integrity without disturbing the microbiological environment [15]. For postmenopausal women, prolonged-release hyaluronic acid formulations have shown therapeutic outcomes comparable to estrogen therapy, but without systemic hormonal effects [15]. These findings highlight the potential for biologic and polymer-based therapies to offer safe, sustained improvements in sexual comfort and tissue quality.

Future mechanistic studies should integrate histology, imaging, microvascular indices, collagen composition, cellular proliferation markers, and genomic profiling to illuminate how restorative changes occur within the H-zone. Understanding the roles of growth-factor signaling, ECM remodeling, and neurotrophic pathways will strengthen clinical translation and help refine biologic formulations to achieve reproducible outcomes.

## Conclusions

Sexual satisfaction is an important component of female psychological and relational well-being, yet the historical focus on the G-spot as a discrete anatomical structure remains scientifically controversial. Accordingly, the H-zone should be interpreted as a proposed conceptual framework for integrating distributed anterior vaginal wall sensitivity, not as an established anatomical structure. Available anatomical and sensory-mapping evidence suggests that the anterior vaginal wall may function as a broader, highly innervated, and vascularized region involved in sexual responsiveness rather than as a single erogenous point. The proposed H-zone framework provides a hypothesis-generating model that integrates anatomical, neurovascular, mechanosensory, and regenerative-biologic concepts. Traditional focal hyaluronic acid augmentation primarily produces mechanical tissue projection, whereas regenerative biologic therapies such as platelet-rich plasma, extracellular vesicles, and stem cell-derived factors may act through molecular pathways related to angiogenesis, epithelial integrity, extracellular matrix remodeling, lubrication, and sensory responsiveness. However, these interpretations are based on a synthesis of existing literature and mechanistic plausibility rather than definitive clinical validation. Well-designed randomized controlled trials, standardized treatment protocols, objective tissue-level assessments, and long-term follow-up studies are required to determine the safety, efficacy, durability, and clinical relevance of H-zone-oriented regenerative therapy.
